# Luminescent Ln-Ionic Liquids beyond Europium

**DOI:** 10.3390/molecules26164834

**Published:** 2021-08-10

**Authors:** Cláudia C. L. Pereira, José M. Carretas, Bernardo Monteiro, João P. Leal

**Affiliations:** 1 LAQV-REQUIMTE, Department of Chemistry, NOVA School of Science and Technology, Universidade Nova de Lisboa, 2829-516 Caparica, Portugal; ccl.pereira@fct.unl.pt; 2 Centro de Química Estrutural (CQE), Departamento de Engenharia e Ciências Nucleares (DECN), Campus Tecnológico e Nuclear, Instituto Superior Técnico, Universidade de Lisboa, Estrada Nacional 10, 2695-066 Bobadela, Portugal; carretas@ctn.tecnico.ulisboa.pt; 3 Centro de Química Estrutural (CQE), Departamento de Engenharia Química (DEQ), Campus Tecnológico e Nuclear, Instituto Superior Técnico, Universidade de Lisboa, Estrada Nacional 10, 2695-066 Bobadela, Portugal

**Keywords:** lanthanides, ionic liquids, luminescence, spectroscopy, NIR

## Abstract

Searching in the Web of Knowledge for “ionic liquids” AND “luminescence” AND “lanthanide”, around 260 entries can be found, of which a considerable number refer solely or primarily to europium (90%, ~234). Europium has been deemed the best lanthanide for luminescent applications, mainly due to its efficiency in sensitization, longest decay times, and the ability to use its luminescence spectra to probe the coordination geometry around the metal. The remaining lanthanides can also be of crucial importance due to their different colors, sensitivity, and capability as probes. In this manuscript, we intend to shed some light on the existing published work on the remaining lanthanides. In some cases, they appear in papers with europium, but frequently in a subordinate position, and in fewer cases then the main protagonist of the study. All of them will be assessed and presented in a concise manner; they will be divided into two main categories: lanthanide compounds dissolved in ionic liquids, and lanthanide-based ionic liquids. Finally, some analysis of future trends is carried out highlighting some future promising fields, such as ionogels.

## 1. Introduction

What is called an ionic liquid (IL) has a very broad definition, comprising multiple substances possessing a wide diversity of structures and properties. An IL consists of both organic and inorganic ions, and may contain more than one cation or anion. Normally, a substance is considered to be an IL if completely composed of ions, with a melting point below 100 °C. Within ILs, there are electrostatic and dispersive interactions at different length scales, leading to a highly anisotropic character. The ions have a large structural diversity which varies from inorganic to organic, simple to complex, including fully or partially ionized acid or base, organic polymeric metal ions, or metalated coordination polymers [1], giving a boundless variety of cation/anion combinations, estimated around the order of 10^19^ [2].

The first IL (ethylammonium nitrate) was reported in 1914 by Paul Walden, who never expected that ILs would become such an important scientific area a century later. The number of papers on ISI Web published about ILs in the last five years is more than 38,000; this is explained by several factors: ILs are environmentally friendly solvents with properties such as extremely low vapor pressure, low combustibility, excellent thermal stability, and a wide temperature range in their liquid state. The low volatility and combustibility of ILs, along with the possibility of building ionic liquids in which physical and chemical properties can be fine-tuned, has been a reason for their enormous use in recent years [3,4,5]. They have been extensively used as low-environmental-impact solvents in catalysis and separation techniques, as lubricants and additives, as auxiliaries in analytical techniques, as thermal fluid, and as electrolytes [6,7,8,9,10,11,12,13,14]. Additionally, a wide variety of ILs are transparent through the visible and NIR (near-infrared) spectral regions—a key property for optical solvents [15]—and today new ILs are designed for specialized applications in optics and soft luminescent materials [16].

**Figure 1:**
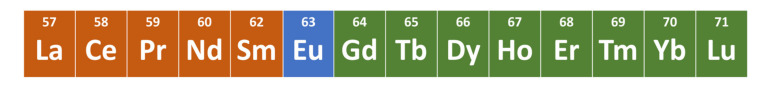
Lanthanide family, excluding Promethium (Pm with atomic number 61 does not exist in nature). Eu (not covered in this paper) is in blue; lighter elements in brown and heavier elements in green.

Lanthanides (Ln) are a group of 15 chemical elements, with atomic numbers 57 through 71, all of which have one valence electron in the 5d shell. All of these elements present similar chemical properties, with +3 being the most stable oxidation state, but the +2 and +4 oxidation states are also common [17] (Figure 1). 

Most lanthanides present luminescence [18], and this property has been abundantly explored by studying several lanthanide compounds in different chemical environments. The incorporation of metals into ILs opens the potential to combine additional chemical and physical properties in the ILs. In this light, Ln cations are very interesting for their potential to endow ILs with additional features, as Ln compounds are well known for their outstanding properties, including optical, magnetic, or catalytic activities. Focusing on the luminescent properties of the Ln^3+^ cations, they present unique optical features over other luminophores, such as excellent luminescence efficiency, tunable emission (choice of Ln^3+^), large pseudo-Stokes shifts, long-lived excited states, and unique monochromaticity [19]. As such, it is not surprising that Ln compounds have been developed for light amplification and generation, security tags, drug delivery, bioimaging, luminescent sensors, and solar energy conversion, among many other applications [20]. The luminescence of Ln^3+^ ions can arise from intraconfigurational 4f–4f transitions, interconfigurational 4f^n^→4f^n^5d^1^ transitions, and charge–transfer transitions (ligand to metal and metal to ligand). The emission of Ln^3+^ ions derived from f–f transition covers the NIR (Pr^3+^, Nd^3+^, Sm^3+^+, Eu^3+^, Dy^3+^, Ho^3+^, Er^3+^, Tm^3+^, Yb^3+^) through visible (Pr^3+^, Nd^3+^, Sm^3+^, Eu^3+^, Tb^3+^, Dy^3+^, Ho^3+^, Er^3+^, Tm^3+^) to UV (Gd^3+^) regions.

**Figure 2:**
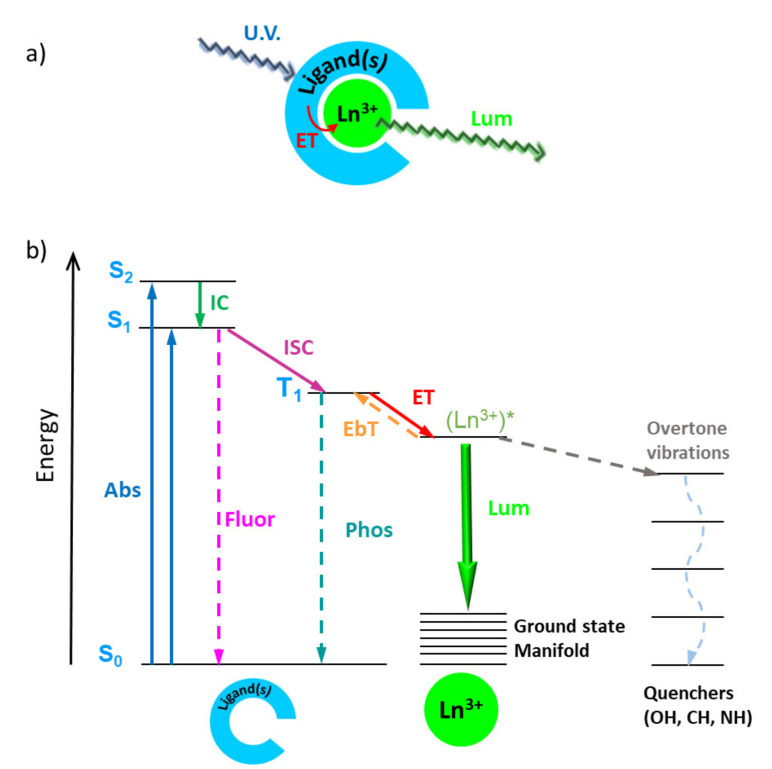
(**a**) Schematic representation of the antenna effect, and (**b**) a Jablonski diagram of the antenna effect (solid arrows) together with competing energy transitions (dashed arrows). Abs: absorbance; IC: internal conversion; Fluor: fluorescence; ISC: intersystem crossing; Phos: phosphorescence; ET: energy transfer; EbT: energy back-transfer; Lum: luminescence.

It has long been known that the molar absorptivity of lanthanide ions is very low (ε = 2–12 M^−1^cm^−1^), but this problem can be easily addressed using intramolecular energy transfer processes commonly known as the “antenna effect”. The “antenna effect” is a concept introduced by S. I. Weissman in the early 1940s [21], which refers the use of highly absorptive organic ligands coordinated with Ln ions that function as light harvesters. The mechanism consists of intramolecular energy transfer from excited states of the ligands to the Ln ion, which dramatically enhances Ln’s luminescence, even under low excitation energy. A detailed explanation of the sensitization of Ln ions is beyond the scope of this manuscript, but there are many publications addressing this issue that an interested reader can consult [22,23]. Figure 2 presents a schematic representation of the antenna effect, as well as its Jablonski diagram including other competing energy transitions within the phosphor molecule.

**Figure 3:**
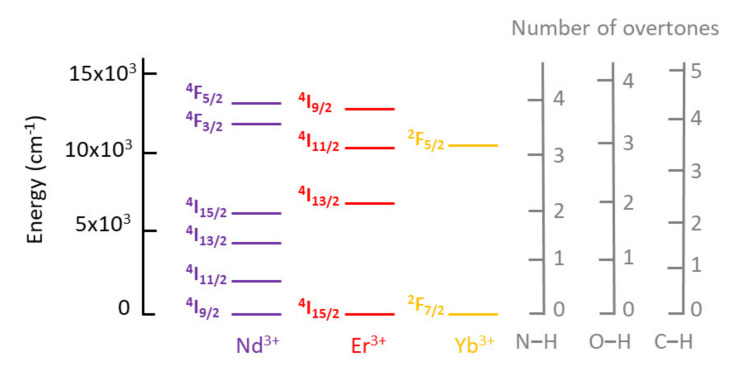
Energy diagram of the NIR luminescent states of Nd^3+^, Er^3+^, and Yb^3+^, and C–H, N–H, and O–H overtones.

A major problem in Ln emission is luminescence quenching by energy loss through non-radiative relaxation pathways, due to energy transfer to high-energy local-mode vibrations such C–H, N–H or O–H stretching modes in coordinating ligands and solvent molecules. This is particularly relevant for NIR luminescent Ln phosphors due to the narrow energy gap between their lowest excited state and highest ground state levels. Hence, the number of vibration quanta required for their non-radiative deactivation is smaller [24]. For instance, for Er^3+^ compounds, two activated O–H vibrations are enough to enable a radiationless return to the ground state. In the case of Ho^3+^-ILs, only one example has been reported to date, by Mudring et al. [2]. Thus, it is not surprising that NIR luminescence is more sensitive to quenching by water than the visible luminescence of Eu^3+^ [25]. For a better visualization, Figure 3 presents an energy diagram of the NIR luminescent states of Nd^3+^, Er^3+^, and Yb^3+^, with varying numbers of C–H, N–H, and O–H overtones.

In 2010, a review by Eliseeva and Bünzli made a point of the utility of lanthanide luminescence for a variety of functional materials, including “soft” luminescent materials such as liquid crystals, ionic liquids, and ionogels [26]. A review of lanthanides and ionic liquids, published in 2010 by Mudring and Tang [27], addressed various topics such as catalysis and luminescence. In a 2013 review, Feng and Zhang approached hybrid compounds by analyzing various properties including the luminescence of compounds containing Ln ions [28]. Additionally, ionic liquids containing lanthanides (but not exclusively) have been studied for their magnetic properties [29]. Recently, Prodius and Mudring conducted a review discussing the structural and coordination chemistry of ILs derived from rare earth metals, and their practical applications [20].

The combination of Ln with ILs has risen as a powerful platform for potential application in several hi-tech processes, screen display technologies, anticounterfeiting technology, heat-storage materials, in situ imaging, optical sensing, energy harvesting, advanced luminescent coatings, etc. [30,31].

Due to its outstanding emissive properties, europium has received special attention in optical studies based on its luminescence [32,33,34,35,36,37]. In this review, the focus will be on the luminescence of ionic liquids/lanthanides other than europium, to which much less attention has been given.

The application of ILs in lanthanide chemistry has raised much interest in recent years. Their initial use was as a simple solvent, but they have been playing an increasing role not only as solvents, but also as reagents, templates, binders, linkers, or property modifiers. The following subsections will address several of these roles in the combination of lanthanides with ionic liquids.

## 2. Luminescent Ln Dissolved in Ionic Liquids (Ln@ILs)

In 2004, Binnemans et al. presented ILs as promising solvents for NIR-emitting lanthanide complexes, due to their properties as polar non-coordinating solvents, capable of solubilizing a large number of Ln complexes [25]. They dissolved Nd^3+^-tosylate, -bromide, -triflate and -sulfonylimide complexes in 1-alkyl-3-methylimidazolium-ILs containing the same anion as the Nd complexes. NIR luminescence spectra of these Nd^3+^-salts were measured by direct excitation of the metal ion. Furthermore, intense NIR luminescence was observed upon ligand excitation of the Nd complexes with 1,10-phenanthroline or β-diketonate ligands.

**Figure 4:**
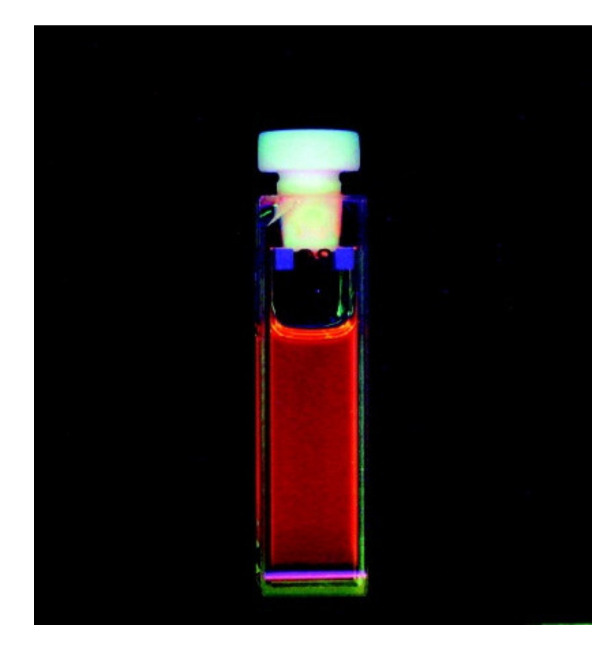
Luminescence of [C_6_mim][Sm(nta)_4_] (nta = 2-naphthoyltrifluoroacetonate) dissolved in the ionic liquid [C_6_mim][Tf_2_N] under UV light (λ = 365 nm) irradiation. Reproduced with permission from [38].

Another manuscript, from the same research group, presents highly luminescent anionic Sm^3+^ *β*-diketonate and dipicolinate complexes when dissolved in the imidazolium ionic liquid [C_6_mim][Tf_2_N] [38]. A judicious choice of the counterion of the Sm^3+^-complex ensured the solubility of the salts in the ionic liquid. Luminescence spectra were recorded for the complexes dissolved in the imidazolium ionic liquid (Figure 4), and compared with the luminescence of the same complexes in acetonitrile or water, showing that [C_6_mim][Tf_2_N] is a better spectroscopic solvent to study Sm^3+^ luminescence. High-luminescence quantum yields were observed for all of the Sm^3+^-*β*-diketonate complexes in IL solutions.

In 2005, Mudring et al. [39] reported the emission spectra of PrI_3_ and Pr(Tf_2_N)_3_ in the ionic liquid [bmpyr][Tf_2_N]. After excitation to the ^3^P_1_ level, remarkable luminescence not only from the ^1^D_2_ level, but also from the ^3^P_0_ and even from the ^3^P_1_ levels, was observed. It is especially noteworthy that both the solutions of PrI_3_ and Pr(Tf_2_N)_3_ in [bmpyr][Tf_2_N] show emissions from the ^3^P_J_ level with unusually high intensities, even at room temperature. It is more common that the non-radiative population of the ^1^D_2_ level from the excited ^3^P_J_ states is preferred at this temperature.

These papers led to a joint publication of two articles in 2005 and 2006; anhydrous NdI_3_ and ErI_3_ were dissolved in carefully dried batches of the [C_12_mim][Tf_2_N] ionic liquid [40]. Binnemans et al. observed an intense NIR emission for both the Nd^3+^ and Er^3+^ ions, provided by the low water content of the ionic liquid. When the content of water in the solution increased, even if only exposed to atmospheric moisture, a rapid decrease in the luminescence intensity was noticed. In another publication, the authors reported the optical properties of Ln^3+^ iodides (Ln = Nd, Dy, Tb) in the ionic liquid [C_12_mim][Tf_2_N] [41]. As expected, the absence of any C–H, N–H, or O–H high-energy oscillators in the immediate neighborhood of the Ln^3+^ ions contributes to excellent luminescence properties, because non-radiative decay becomes less likely when compared to typical solvents.

In another 2005 study, Bünzli et al. used the IL 1-dodecyl-3-methylimidazole chloride, [C_12_mim]Cl, and doped it with 1 mol-% of the Ln ternary complexes [Ln(tta)_3_(phen)] (Ln = Nd, Er, Yb) [42], obtaining luminescence at room temperature. The spatial arrangement around the lanthanide metal was very similar in both the mesomorphic sample and the parent *β*-diketonate complex to that in the europium sample. Moreover, the mesomorphic samples containing Nd, Er, and Yb showed relatively intense NIR luminescence.

Ionic liquids were used by Binnemans et al. [43] as solvents for dispersing luminescent lanthanide-doped LaF_3_:Nd^3+^ nanocrystals with a stabilizing ligand (betaine = N,N,N-trimethylglycine). LaF_3_:Nd^3+^:betaine could successfully be dispersed in [C_4_mpyr][Tf_2_N], [C_4_mpyr][TfO], and [C_4_mim][Tf_2_N]. NIR luminescence was observed for the Nd^3+^-based systems.

Highly luminescent anionic Sm^3+^–diketonate and –dipicolinate complexes were dissolved in the imidazolium ionic liquid [C_6_mim][Tf_2_N]. The Sm^3+^ complexes considered by Binnemans et al. [38] were [C_6_mim][Sm(tta)_4_], [C_6_mim][Sm(nta)_4_], [C_6_mim][Sm(hfa)_4_], and [choline]_3_[Sm(dpa)_3_]. Luminescence spectra were recorded for the Sm^3+^ complexes dissolved in the imidazolium ionic liquid, as well as in a conventional solvent. These experiments demonstrate that [C_6_mim][Tf_2_N] is a suitable spectroscopic solvent for studying Sm^3+^ luminescence. High-luminescence quantum yields were observed for the Sm^3+^–diketonate complexes in solution.

These years provide a series of studies by several research groups. Gatsis and Mudring [44] reported on C_12_mimBr, a well-known ionic liquid crystal, doped with SmBr_3_, TbBr_3_, and DyBr_3_, which allowed them to obtain ionic liquid crystal materials that show luminescence in the three basic colors (red, green, and orange). Most interestingly, the emission color for the TbBr_3_- and DyBr_3_-containing materials can be tuned from bluish white (mainly [C_12_mim][Br] emission) to green (for TbBr_3_) or orange-yellow (for DyBr_3_), depending on the wavelength of the excitation light used.

The luminescence properties of TbCl_3_(phen)_2_(H_2_O)_3_ in the solid state and in solutions of the [C_12_mim][Cl] ionic liquid were investigated by Puntus et al. [45]. Luminescence data contributed to elucidating the structural peculiarities of lanthanide chlorides, and revealed a highly efficient luminescence for the Tb^3+^ complex.

Luminescent soft materials were obtained by Huarong et al. [46] by dissolution of lanthanide (Nd, Er) oxides and organic ligands (tta, phen) into carboxyl-functionalized ionic liquids (IL1 = 3-(5-carboxypropyl)-1-methylimidazolium bromide; IL2 = 3-(5-carboxy propyl)-1-butylimidazolium bromide). Optical properties of the soft materials, such as color and luminescence, can be adapted by simply changing the type of lanthanide ions and/or addition of an organic ligand. The obtained ionic liquids (Nd-tta/IL2, Er-tta/IL-2) present luminescence in the NIR region, and their excitation and emission spectra are very similar to those of Nd-tta/IL-1 and Er-tta/IL-1.

The luminescent properties of Tb^3+^ dissolved in ionic liquids were studied by Hopkins and Goldey [47]; this study included a simple lanthanide compound (TbCl_3_) dissolved in a [C_4_mim][Br]/water mixture, and showed that the [C_4_mim][Br] ionic liquid does sensitize Tb^3+^ luminescence. 

The emission properties, including luminescence lifetimes, of the lanthanide complexes Ln(Tf_2_N)_3_ (Ln^3+^ = Pr, Nd, Sm, Dy, Er, Tm) in the ionic liquid [bmpyr][Tf_2_N] were presented by Brandner et al. [48]. The luminescence lifetimes in these systems are remarkably long compared to values typically reported for Ln^3+^ complexes in solution, reflecting weak vibrational quenching.

1-Butyl-3-methylimidazolium benzoate-IL, [C_4_mim][BA] was found by Viswanathan et al. [49] to enhance the fluorescence of Tb^3+^; this enhancement resulted from a sensitization of the lanthanide fluorescence by the benzoate anion of the IL, and a reduction in the non-radiative channels provided by the non-aqueous environment caused by the NIR. The authors also found that the fluorescence enhancement of the lanthanides in the IL was limited due to an inner filter effect, which resulted from strong benzoate absorption. For Tb^3+^, the strong emission of the ionic liquid in the region 450–580 nm masked the lanthanide emission. To observe the long-lived Tb^3+^ emission and distinguish it from the short-lived emission from the IL, an appropriate delay was used in the detection.

Mudring et al. [50] report the synthesis of interesting new materials such [C_12_mim][Br] and [C_12_mpyr][Br], both doped with TbBr_3_, as these are able to form mesophases over a wide temperature range. All materials show strong green luminescence from the ^5^D_4_ level of Tb^3+^ after excitation into the 4f^8^ → 4f^7^5d^1^ transition. In the case of the imidazolium compound, the color of this emission can be switched between green and blue-white depending on the excitation energy. After excitation with λ_ex_ = 254 nm, strong green emission is observed—mainly from the ^5^D_4_-level of Tb^3+^—while with λ_ex_ = 366 nm, only the blue-white luminescence from the imidazolium cation itself is detected.

Li et al. [51] reported the synthesis of carboxylic-acid-functionalized ILs with linear alkyl chains of various lengths on the cations ([Carb-C_n_mim]Br, n = 8, 12, 16); they also tested the solubilizing capability of the synthesized ILs relative to Tb_4_O_7_, which led to soft luminescent materials combining Ln^3+^ ions and ILs. The luminescent properties of the obtained materials were investigated.

In 2014, Bortoluzzi et al. [52] prepared the ionic liquid [P_8,8,8,1_][BrMA] via the addition of HBrMA to [P_8,8,8,1_][CH_3_OCO_2_]. The doped ionic liquid Tb@[P_8,8,8,1_][BrMA] was obtained by adding anhydrous TbCl_3_ to [P_8,8,8,1_][BrMA]. In addition to the measurements performed on the pure complex, the photoluminescence of Tb@[P_8,8,8,1_][BrMA] was investigated, but the emissions from the metal ions were almost completely masked by an intense and broad band covering the range 400–750 nm, which can be mainly attributed to the fluorescence of the ionic liquid.

Highly luminescent tetrakis Sm^3+^ complexes with the dbm ligand and the phosphonium [P_8,8,8,1_]^+^ counterion were synthesized by Malba et al. [53]. Crystal data from [Sm(dbm)_4_][P_8,8,8,1_] show that the Sm^3+^ ions are surrounded by four dbm ligands coordinating, as expected, in a bidentate fashion, efficiently shielding the metal center from solvent molecules. The photoluminescence of both complexes was studied in the solid state, [P_8,8,8,1_][Tf_2_N]-IL, and acetonitrile; as expected, they presented broadening of the photoluminescence emission peaks when passing from the solid state to the complex dissolved in either the IL or a molecular solvent, due to collisions and electrostatic interactions with solvent molecules. The emission level was ^4^G_5/2_ for all Sm^3+^ emissions. The polarizability of the complex determined by the ratio between the integrated areas of the ^4^G_5/2_ → ^6^H_9/2_ and ^4^G_5/2_ → ^6^H_5/2_ transitions, and of the emission spectrum in solid state, was 13.3, which is quite high for a Sm^3+^ complex, and was similar to that of Eu^3+^–β-diketonate complexes. Time-resolved analysis of [Sm(dbm)_4_][P_8,8,8,1_] presented lifetime values of 63.5 μs, 19.1 μs, and 3.1 μs in the solid state, [P_8,8,8,1_][ Tf_2_N]-IL, and acetonitrile, respectively. Internal quantum efficiencies of ~2% in solid state, 1% in IL, and 0.1% in acetonitrile indicate an important contribution of non-radiative recombination pathways in solution.

**Figure 5:**
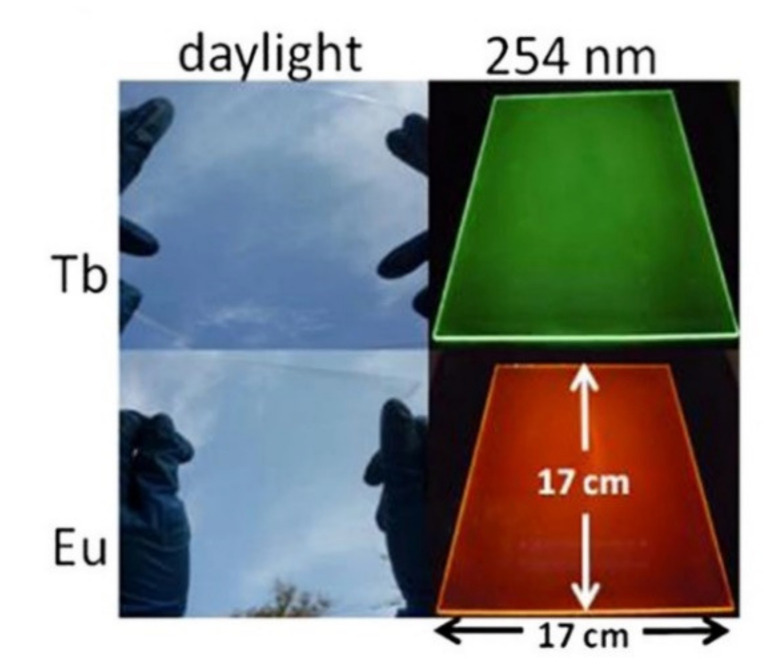
Photographs of the flexible, transparent, luminescent PMMA films (17 × 17 cm^2^) under daylight and a 254-nm UV lamp. Adapted with permission from [54].

Ln^3+^-doped ionic liquids were prepared by dissolving the complex Tb(pybox)_3_ into a bidentate organophosphine-functionalized ionic liquid (1,3-bis-[3-(diphenylphosphinyl)propyl]imidazole bis(trifluoromethylsulfonyl)imide) by Li et al. [54]. These materials show improved luminescence efficiency, attributed to the coordination of ionic liquids with Ln^3+^ ions, and can be beneficial for enhancing the photovoltaic energy conversion efficiency of silicon-based solar cells. The authors prepared large-area (17 × 17 cm^2^) flexible, transparent, luminescent poly(methyl methacrylate) thin films (Figure 5), and applied them as luminescent coatings to the silicon-based heterojunction solar cells, obtaining—in the best case, using the Tb^3+^-containing film—an increase in performance of ~16%.

**Figure 6:**
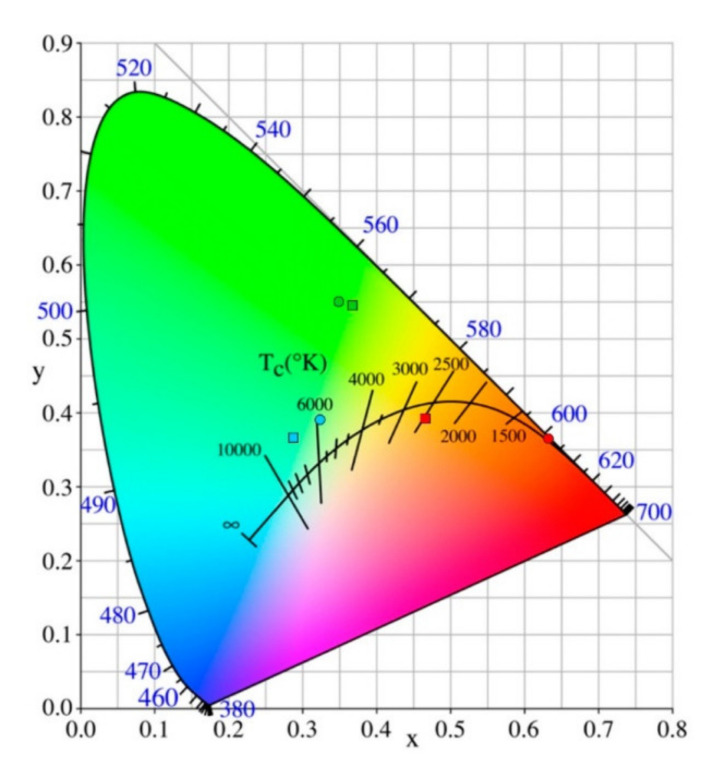
CIE 1931 chromaticity diagram for Ln compounds (red, (Eu^3+^); green, (Tb^3+^); blue, (Dy^3+^); circle: at RT, and square: at LT (77 K)). Reproduced with permission from [55].

Recently, Tang et al. synthesized complexes of La, Nd, Eu, Tb, Dy, and Yb with dicyanamide (DCA) ions—[C_2_mim][Ln(DCA)_4_(H_2_O)_4_]—using a DCA-based ionic liquid [55]. Luminescence studies were conducted with Eu, Tb, and Dy compounds at room (RT) and liquid nitrogen (LT) temperatures, with the spectra presenting the corresponding characteristic f−f transitions. The emission spectra of the Tb compound were similar to the analogous spectra of the Tb[N(CN)_2_]_3_ and Tb[N(CN)_2_]_3_·2H_2_O, although with different relative intensities, which is an indication of different local symmetries around the Tb^3+^ center. The emission lifetimes (^5^D_4_) increased from 0.60 ms at RT to 0.71 ms at LT. In the case of the Dy compound, hypersensitive transition (Δ*L* = 2, Δ*J* = 2) ^4^F_9/2_ → ^6^H_13/2_ was the most intense and, thus, responsible for the yellowish luminescence. The Dy^3+^ (^4^F_9/2_) decay time of 11.9 μs is comparable to that of the series [C_6_mim]_5−x_[Dy(SCN)_8−x_(H_2_O)_x_] (x = 0−2) [56], which will be discussed in the Ln-ILs section. The CIE coordinates (chromaticity coordinates—CIE color space is a quantitative link between distributions of wavelengths in the electromagnetic visible spectrum and physiologically perceived colors in human color vision) of the Ln compounds were determined from the respective RT and LT emission spectra, showing a great variety of colors from the red (Eu) to the green-yellow (Tb) and blue-yellow (Dy) regions (Figure 6).

Table 1 presents a list of the Ln@ILs discussed in this review, ordered by Ln^3+^ center along with their lifetimes and associated excited level and, when available, absolute quantum yield.

## 3. Luminescent Ln-based Ionic Liquids (Ln-ILs)

The dissolution of Ln salts within ILs is a neat and simple method, but when the anionic moieties of the ILs have no coordinating capability, this method only enables low concentrations of Ln ions due to the low solubility of the salts. However, when an IL with an anionic part has some coordinating ability, higher concentrations can be achieved due the formation of Ln-based ionic liquids (Ln-ILs), in which the Ln ions become part of the ILs in the form of an anionic complex.

In 2003, Jensen et al. studied the stoichiometry of Eu and Nd complexes with the Htta ligand in a biphasic aqueous/[C_4_mim][Tf_2_N] system [57]. All of the characterization techniques supported the formation of anionic [Ln(tta)_4_]^-^ species with no water coordinated to the metal center in the [C_4_mim][Tf_2_N]-RTIL phase, instead of the hydrated neutral complexes—Ln(tta)_3_(H_2_O)_n_—that form in the nonpolar molecular solvents xylene or chloroform. The presence of anionic Ln complexes in the IL is made possible by the exchange of [Tf_2_N]^-^ anions into the aqueous phase in exchange for the [Ln(tta)_4_]^-^ complex. Additionally, it was shown that the resulting [C_4_mim][Ln(tta)_4_] ion pairs exert little influence on the structure of the ionic liquid phase.

Although Ln-ILs can be formed by the dissolution of Ln salts in coordinating ILs, another strategy is to prepare them from the start.

The first Ln-IL series was published in 2006 by Nockemann et al. [58], based on the [C_2_mim]^+^ cation and lanthanide thiocyanate anions. The Ln-ionic liquids, prepared via a metathesis procedure, presented the general formula [C_2_mim]_x-3_[Ln(NCS)_x_(H_2_O)_y_] (x = 6, Y = 2 (Y); x = 7, Y = 1 (La, Pr, Nd, Sm, Gd, Tb, Ho, Er, and Yb; x = 8, y = 0 (La)), and the luminescence properties of the Sm-ILs were studied a few years later by Ohaion et al. [59]. In this study, the plot of the luminescence intensities of Sm^3+^ solutions vs. NCS/Ln ratios showed that increasing the NSC/Ln ratio leads to detaching of water molecules from the Sm^3+^ center, with their replacement by thiocyanate ligands within the coordination sphere. This result supported the composition proposed by the authors—[C_2_mim]_x-3_[Ln(NCS)_x_(H_2_O)_8-x_]—which assumed an absence of water for NSC/Ln ratios above 8.

The first studies concerning the emission of Ln-ILs were reported by Mudring et al. in two consecutive manuscripts in 2008. The first publication concerned the low-melting-point Eu-ILs [R]_x_[Eu(Tf_2_N)_3+x_] (x = 1 for R = C_3_mim and C_4_mim; x = 2 for C_4_mpyr) [34], while the second concerned the first examples of room temperature ILs (RTILs) that combine magnetic and luminescent properties, by describing the magneto-optical properties of the [C_6_mim]_5-x_[Dy(SCN)_8-x_(H_2_O)_x_](x = 0–2) compounds [56]. All three orange-colored Dy-ILs presented a strong response to a commercial neodymium magnet, and an intense yellow emission characteristic of the Dy^3+^ center. The most intense transition, between the ^4^F_9/2_ and ^6^H_13/2_ levels, presented an extremely sharp line shape, indicating high color purity. For the three Dy-ILs, only one Dy^3+^ is present in the anhydrous [C_6_mim]_5_[Dy(SCN)_8_] analog presenting the highest decay time, explained by the fact that the thiocyanate ligands, unlike water molecules, are not prone to take up the energy of the excited state, thus providing a fairly rigid ligand environment.

In the following year, Getsis et al. reported Dy-based ionic liquid crystals based on the [C_12_mim]_3_[DyBr_6_] compound [60]. This Dy-IL showed interesting luminescent properties together with mesomorphic and superparamagnetic behavior. The [C_12_mim]_3_[DyBr_6_] presented either a bright white or orange yellow emission, depending on the chosen wavelength of excitation. Irradiation with a wavelength of λ_ex_ = 366 nm leads to a bluish-white luminescence characteristic of the imidazolium moiety, while upon irradiation with a wavelength of λ_ex_ = 254 nm, the compound turns orange due to the Dy^3+^ ion emission. By comparing the luminescence spectra of the [C_12_mim]_3_[DyBr_6_] and pure [C_12_mim]Br, it becomes evident that the bluish-white appearance of the Dy-IL comes from a combination of the luminescence of the [C_12_mim]^+^ cations and a small contribution from the Dy^3+^ transition around 480 nm. It is also interesting that the lifetime of the emission of the most intense transition (^4^F_9/2_ → ^6^H_13/2_) was unaffected by temperature or by the physical state of the compound.

The same group extended these studies to the Tb^3+^ analog and the [C_12_mpyr]^-^ anion [51]. To do so, they prepared two new Tb-ILs—[C_12_mim]_3_[TbBr_6_] and [C_12_mpyr]_3_[TbBr_6_]—as well as two samples consisting of [C_12_mim]Br and [C_12_mpyr]Br ILs, both doped with TbBr_3_. All four samples present mesomorphic behavior, and are capable of forming smectic liquid crystalline phases. The Tb-doped ILs crystallize around room temperature, while the neat Tb-ILs solidify as liquid crystal glasses around –5 °C. All compounds present strong green luminescence with the typical Tb^3+^ emission bands, with long lifetimes of the excited ^5^D_4_ level. The pyrrolidinium compounds had somewhat higher lifetimes in comparison with the imidazolium analogs, with the latter presenting a more intense luminescence emission due to an energy transfer from the imidazolium cation to the Tb^3+^ ion, as was reported in the case of [C_12_mim]_3_[DyBr_6_], as described above [60]. Additionally, just like in the case of the Dy-IL with the [C_12_mim]^-^ anion, it is possible to tune the color emission of the Tb samples with this imidazolium between green and white by using a UV light excitation of 254 nm or 366 nm, respectively.

**Figure 7:**
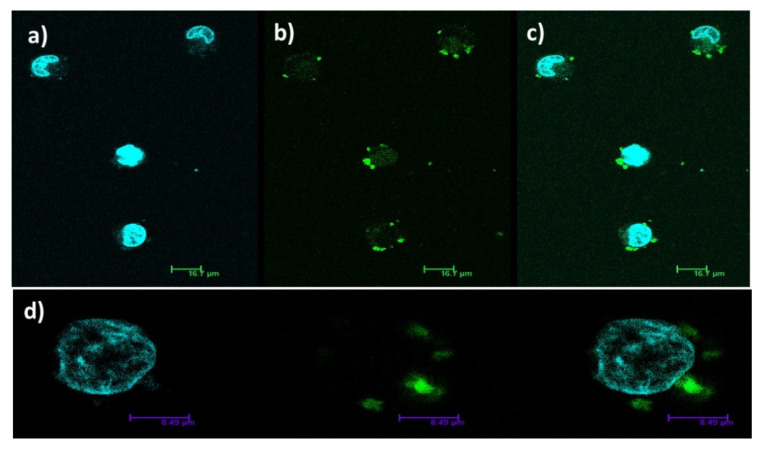
Visualization of live cancer cells in the presence of [Ph_4_P]_5_[Dy(SCN)_8_] by confocal laser microscopy: (**a**) localization of the nucleus (control test showing cell nucleus without using Dy-IL); (**b**) localization of the Dy-IL; (**c**) (a) and (b) overlapped; (**d**) demonstration of how Dy-IL was taken by the cells up (λ_ex_ = 458 nm). Reproduced with permission from [61].

Li et al. reported a series of four multifunctional [Dy(SCN)_8_]-ILs using different phosphonium cations with luminescence, paramagnetism, and tumor mitochondrial targeting properties: [Ph_4_P]_5_[Dy(SCN)_8_], [Ph_3_PBnOEt]_5_[Dy(SCN)_8_], [Ph_3_PBnNO_2_]_5_[Dy(SCN)_8_], and [Ph_3_PBn]_5_[Dy(SCN)_8_] (Bn = benzyl group) [61]. These Dy-ILs were studied as fluorescence imaging markers in vital cell cultures via confocal laser microscopy (Figure 7). It was found that the uptake of these lipophilic Dy-ILs occurred in the cell membrane, and with selective inhibition of the growth of tumor cells.

In 2015, Tang et al. reported five hexanitratosamarate(III) salts, of which four were Sm-ILs—[C_n_mim]_3_[Sm(NO_3_)_6_] (n = 2, 4, 6, 8) [62]. The fifth compound—[MC_1_mim]_3_[Sm(NO_3_)_6_] (MC_1_mim = 1,2,3-trimethylimidazolium)—although not an IL, was useful for the structural elucidation of the hexanitratosamarate(III) anion. The three Sm^3+^ excitation states ^4^G_7/2_, ^4^F_3/2,_ and ^4^G_5/2_ could be excited efficiently, but only the emissions from the ^4^G_5/2_ level were observed intensively. A possible explanation is that the two higher excitation states ^4^G_7/2_ and ^4^F_3/2_ are close in energy to the ^4^G_5/2_ level, allowing electrons to relax to the lower level quickly through non-radiative transition processes. The lifetime luminescence of all compounds falls in the 114.4–130.3-μs range in acetonitrile, which is longer than that of most Sm^3+^ compounds. The reason for these high values is the absence of C–H, O–H, or N–H bonds in the [Sm(NO_3_)_6_]^3−^, which would increase non-radiative transitions. In fact, for the [C_6_mim]_3_[Sm(NO_3_)_6_]-IL, the addition of only 50 μL of water to 5 mL of the Sm-IL led to a sharp decrease in the lifetime from 114.8 μs to 3.75 μs.

Han et al. prepared a series of [C_4_mim]_3_[LnCl_6_] (Ln = Sm, Dy, Er, Yb) crystals from solutions of LnCl_3_ dissolved in [C_4_mim][Cl] [63]. Additionally, to study the importance of cross-relaxation within the Sm^3+^ and Dy^3+^ samples, they also prepared two samples of these ions diluted in Gd^3+^ (5% Sm and 5% Dy). From the crystal data, the authors concluded that the first coordination sphere of the Ln^3+^ ions is composed of six Cl^–^ anions, in a slightly distorted octahedral LnCl_6_^3–^ fashion, while the second coordination sphere consists of nine [C_4_mim]^+^ cations. 

**Figure 8:**
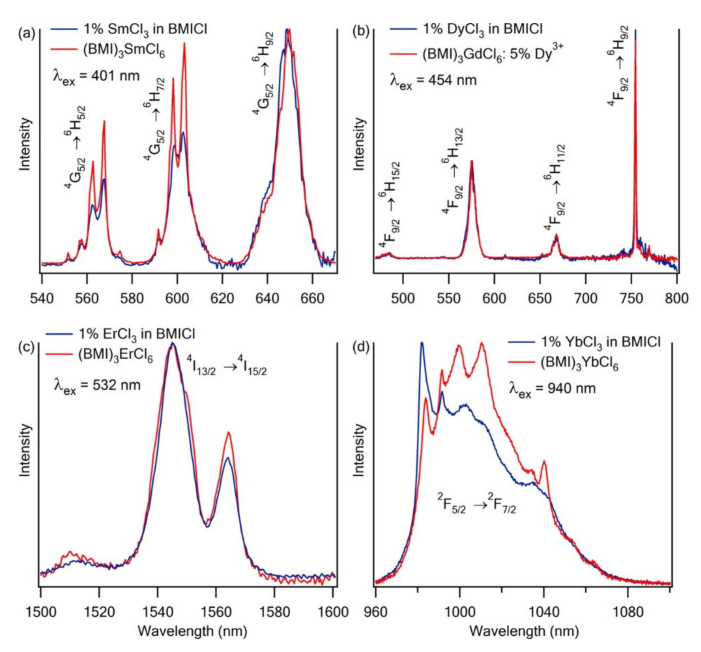
Normalized emission spectra of (**a**) 1% SmCl_3_ in C_4_mimCl and [C_4_mim]_3_[SmCl_6_] crystals; (**b**) 1% DyCl_3_ in C_4_mimCl and [C_4_mim]_3_[GdCl_6_]:[5% Dy^3+^] crystals; (**c**) 1% ErCl_3_ in [C_4_mim][Cl] and [C_4_mim]_3_[ErCl_6_] crystals; and (**d**) 1% YbCl_3_ in [C_4_mim][Cl] and [C_4_mim]_3_[YbCl_6_] crystals. Solution and crystal spectra were measured at 338 K and 278 K, respectively. Reproduced with permission from [63].

The emission spectra and luminescence lifetimes of both [C_4_mim]_3_[LnCl_6_] crystals and LnCl_3_ in [C_4_mim][Cl] solutions were determined in order to study the surroundings of the metals in solution (Figure 8). The spectroscopic similarity found between both spectra suggest that crystalline [C_4_mim]_3_[LnCl_6_] is a good model of the Ln^3+^ coordination environment in [C_4_mim][Cl] solutions.

In this study, it deserves to be highlighted that, for this system, the second-coordination-sphere quenching is relatively efficient. For example, for the small-energy-gap Ln^3+^, the multiphonon relaxation of Ln(Tf_2_N)_3_ complexes in [bmpyr][Tf_2_N] is much less effective [48], notwithstanding the fact that the maximum vibrational energy within the [Ln(Tf_2_N)_x_]^3−x^ is ∼1340 cm^−1^ whilst the phonon cutoff for the LnCl_6_^3-^ is ∼260 cm^−1^. The explanation for this is that the small radius of the first coordination sphere of the [LnCl_6_]^3−^ anions does not provide adequate protection from the high-energy C−H oscillators from the [C_4_mim]^+^ counterions of the second coordination sphere.

**Figure 9:**
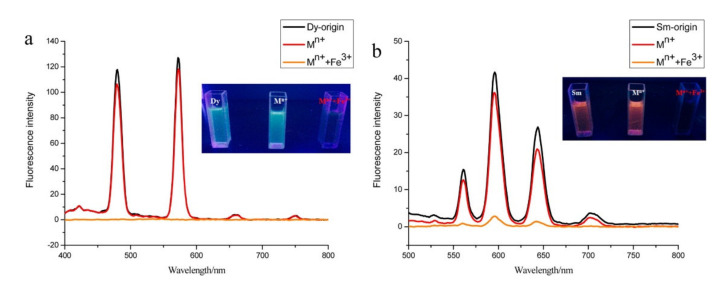
Comparison of the photoluminescence intensity of (**a**) [C_4_mim][Dy(NO_3_)_4_] and (**b**) [C_4_mim][Dy(NO_3_)_4_] mixed with M^n+^ (Ca(II), Al(III), Zn(II), Cu(II), Pb(II), Hg(II), Cd(II), Co(II), Fe(II), Ni(II), and Cr(III)), in the absence and presence of Fe(III). Insets are the corresponding luminescence images under UV light irradiation at 365 nm. Reproduced with permission from [64].

Two new lanthanide-based RTILs—[C_4_mim][Ln(NO_3_)_4_] (Ln = Dy, Sm)—were synthesized and characterized by Fan et al. [64]. The photoluminescence properties of these hydrostable and ecofriendly Ln-ILs were studied at room temperature in deionized water, and their strong fluorescence indicates that these ILs could be used as good luminescent materials. As such, the authors studied their application as fluorescent sensors for Fe(III) (Figure 9). 

Both Ln-ILs presented high specific recognition for aqueous Fe^3+^ ions, even discernible with the naked eye, with no interference by many other common metal ions.

In 2016, Pohako-Esko et al. prepared a series of hexahalocerate(III) salts with the general formula [cation]_3_[CeHal_6_] by dissolving anhydrous cerium trihalides in imidazolium halide ionic liquids [65]. Complexes with different halides—[CeCl_6_]^3−^ and [CeBr_6_]^3^^−^—were combined with [C_4_mim]^+^, [C_6_mim]^+^, [C_8_mim]^+^, and [C_10_mim]^+^ counterions along with the mixed-halide compound [C_4_mim]_3_[CeBr_3_Cl_3_]. The melting points of the synthesized salts varied between 85 °C for [C_10_mim][CeBr_6_] and 155 °C for [C_6_mim][CeCl_6_]. The melting points increase slightly from carbon number n = 4 in the alkyl chain to n = 6, and then continuously decrease with increasing alkyl chain lengths. However, only two of the salts can be considered ILs—[C_10_mim][CeBr_6_] and [C_4_mim]_3_[CeBr_3_Cl_3_]. [C_4_mim][CeHal_6_] salts presented intense photoluminescence ascribed to Ce^3+^-based 5d–4f-centered emission ranging from soft UV to the border of visible emission. The emission was made possible either by direct excitation of the Ce^3+^ center or by the sensitizing effect of the [C_4_mim]^+^ counterion. These results show that emissive Ln-ILs can be designed with parity-enabled Ce^3+^-based luminescence.

Alvarez-Vicente et al. prepared and characterized the [P_66614_][LnCl_6_] and [P_4444_][LnCl_6_] of the entire Ln^3+^ series plus Y^3+^ and Sc^3+^, as well as the [P_4448_][LnCl_6_] analogs for Ln = Ce, Nd, Sm, Tb, Dy, and Er [66]. The entire [P_66614_][LnCl_6_] series, with the longer alkyl chains in the phosphonium, is composed exclusively of room temperature ionic liquids, as commonly found for this cation [67,68,69,70], with melting points (m.p.) between −58 and −40 °C, with the exception of the La^3+^-IL at –1.6 °C. In the case of the [P_4444_][LnCl_6_] series, all compounds are ILs with an m.p. in the range 43–96 °C—again, with the exception of the La^3+^-IL, which has an m.p. of 103 °C. The [P_4448_][LnCl_6_] series showed more irregular behavior and trends than the other series. The lighter lanthanides had an m.p. only slightly lower than the [P_4444_]^+^-analogs; however, upon cooling, these compounds behave like supercooled liquids with rather low crystallization temperatures (between 12 and 18 °C). In the case of the heavier lanthanides, the compounds have relatively low melting points, ranging from −6 to −48 °C. EXAFS measurements with selected samples confirmed the LnCl_6_ coordination in the liquid state, with the Ln···Cl distance decreasing with decreasing Ln ionic radius. The authors performed magnetic, electrochemical, and luminescence studies on selected samples. Concerning the photoluminescence studies, detailed studies were conducted concerning the excitation, emission, and decay times of the visible-light-emitting Tb-, Dy-, and Sm-ILs, as well as the visible-light- and NIR-emitting Nd- and Er-ILs. All transitions in the excitation and emission spectra were assigned, and it is worth highlighting the relatively long luminescence lifetimes of 2.470 μs and 2.725 μs for the [P_66614_][NdCl_6_] and [P_66614_][ErCl_6_], respectively.

Zheng et al. synthesized novel fluorescent RTILs based on Dy^3+^ ([MOEmim][Dy(NO_3_)_4_]) [71]; they exhibited good fluorescence properties with light blue (Dy^3+^) luminescence. This is the first time that the application of Ln-RTILs as fluorescence sensors for aromatic compounds has been studied. The sensor worked via the fluorescence-quenching of the phosphor when in the presence of trace amounts of *o*-(*m*-, *p*-)nitrotoluene. Among the three isomeric nitrotoluenes, *p*-nitrotoluene showed the most significant fluorescence-quenching effect. Furthermore, the two fluorescent ionic liquids demonstrated high selectivity toward nitrotoluene even in the presence of methylbenzene, phenol, chlorobenzene, and aminobenzene. Hence, the selective recognition of nitrotoluene from other aromatic compounds may be used for the analytical detection of explosives.

**Figure 10:**
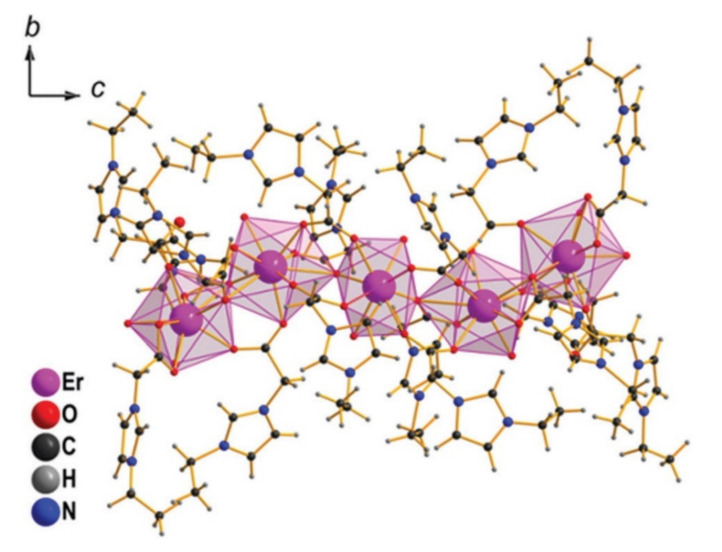
Structure of the [Er_5_(C_2_H_5_-C_3_H_3_N_2_-CH_2_COO)_16_(H_2_O)_8_]^15+^ cation. Reproduced with permission from [2].

When designing ILs, highly charged ions are, by default, ruled out in order to avoid high Coulombic attraction that could easily lead to the compound being solid at room temperature (or below 100 °C). However, in 2017, Prodius et al. published the series [Ln_5_(C_2_H_5_-C_3_H_3_N_2_-CH_2_COO)_16_(H_2_O)_8_](Tf_2_N)_15_ (Ln = Er, Ho, Tm; C_2_H_5_-C_3_H_3_N_2_-CH_2_COO = 1-carboxymethyl-3-ethylimidazolium), featuring the pentanuclear Ln-containing +15 cation [Ln_5_(C_2_H_5_-C_3_H_3_N_2_-CH_2_COO)_16_(H_2_O)_8_]^15+^ (Figure 10) [2].

These ILs were prepared from the reaction of the respective Ln oxide with 1-carboxymethyl-3-ethylimidazolium chloride and LiTf_2_N in water, with the ILs forming a separate phase. These ionic liquids show a low tendency to crystallize, taking a few months for the Er-ILs and Ho-IL to form crystals under ambient conditions. In the case of the Tm-IL, all crystallization attempts failed, and only glass transitions were observed. NIR luminescence studies performed at room temperature showed a broad band at 1540 nm for the Er-IL, with four shoulders assigned to the ^4^I_13/2_ → ^4^I_15/2_ transition of the Er^3+^ ion with an emission lifetime of 0.6 μs. In the case of the Ho-IL, the emission spectra presented a set of bands assign to the ^5^F_5_ → ^5^I_7_, ^5^I_6_ → ^5^I_8_ and ^5^F_5_ → ^5^I_6_ transitions of the Ho^3+^ ion with an emission lifetime of 0.8 μs. The values of the lifetimes are appreciably high for compounds in the liquid state. Usually, Ln^3+^ ions surrounded by water molecules and organic ligands present lifetimes in the ns range. These lifetimes are similar to those found for glasses and complexes surrounded by rigid ligands. Additionally, these Ln-ILs presented the highest ever reported values of the effective moments for magnetic ionic liquids, and extraordinary catalytic activity in the three-component synthesis of ethyl 2-methyl-4-(2-oxo-2,3-dihydro-1H-3-indolyl)-5-phenyl-1H-3-pyrrolecarboxylate.

**Figure 11:**
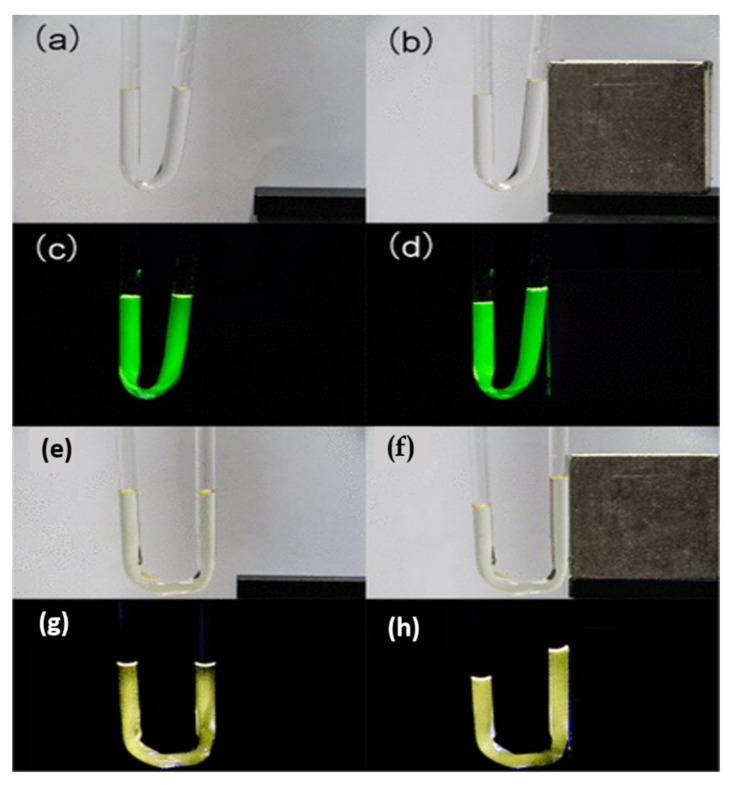
Images of a U-tube filled with Tb-ILs (**a–d**) and Dy-IL (**e–h**), with (**b**,**d**,**f**,**h**) and without (**a**,**c**,**e**,**g**) a NdFeB magnet on the side under the radiation of bright light (**a**,**b**,**e**,**f**) and 365 nm UV light (**c**,**d**,**g**,**h**). Reproduced with permission from [30].

One year later, Cheng et al. reported a series of anhydrous fluorescigenic magnetofluids based on Ln^3+^ ions [RC_n_mim]_2_[Ln(NO_3_)_5_] (Ln = Gd, Tb, Dy; R = H or methyl and n = 2, 4, 6, 8) (Figure 11); all of these compounds are RTILs except for R = methyl with n = 1 [30]. The coordination environment composed of nitrate ligands weakened the Ln^3+^−Ln^3^+ energy transition, thus avoiding concentration-quenching effects. Additionally, there was no relevant energy exchange between the imidazolium counterions and the Ln^3+^ centers. The lifetime value of the Tb-IL series varied between 1.262 and 1.294 ms, while the lifetimes of the Dy-ILs varied from 54.75 to 59.27 μs. The fluctuation of the lifetime values within the different series—i.e., with different imidazolium cations—was explained by the interplay of the influences of the different spacing of the anions. The long luminescence lifetimes of the Ln-ILs confirm that these possess a favorable environment that can protect the Ln^3+^ phosphors from any interference, provided that the structures are integrated. 

**Figure 12:**
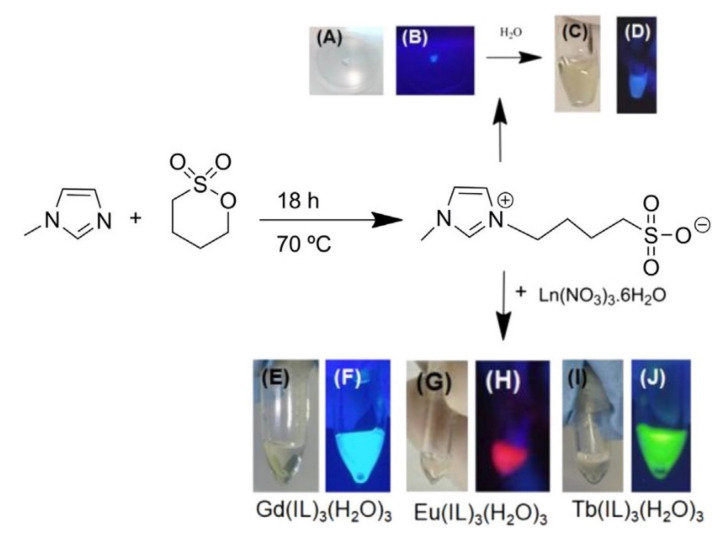
(**A**–**D**) Photographs of ionic liquid 1,4-methylimidazolylbutane sulfonate bromide (white emission) and Ln-ILs: (**E**,**F**) Gd(IL-1)_3_(H_2_O)_3_, (**G**,**H**) Eu(IL-1)_3_(H_2_O)_3_ (red emission), and (**I**,**J**) Gd(IL-1)_3_(H_2_O)_3_ (green emission) under UV light (365 nm) and daylight. Reproduced with permission from [31].

In 2019, Ramos et al. reported the synthesis of the new organic ionic liquid 1,4-methylimidazolylbutane sulfonate bromide (IL-1), and used it to prepare Ln-ILs Ln(IL-1)_3_(H_2_O)_3_ (Ln = Gd, Eu, Tb) (Figure 12) [31]. The triplet level of the organic IL is ~25,000 cm^−1^, and the singlet is ~30,000 cm^−1^. The first excitation state of the Gd^3+^ ion (^6^P_7/2_) is ~32,000 cm^−1^ so, as expected, the Gd-IL only showed ligand photoluminescence. As for the Tb^3+^- and Eu^3+^-ILs, the ligand proved to be more efficient in the sensitization of the Tb^3+^. Furthermore, the absence of a broad phosphorescence emission band in the range 400–600 nm arising from the ligand indicates that the intramolecular energy transfer from the ligand to the Tb^3+^ center was very efficient. The lifetimes determined for the Tb-IL were 0.7712 and 0.6988 ms, for direct Tb^3+^ excitation (370 nm) and ligand excitation (350 nm), respectively. These long lifetime values were the highest reported amongst all Tb soft materials reported.

**Figure 13:**
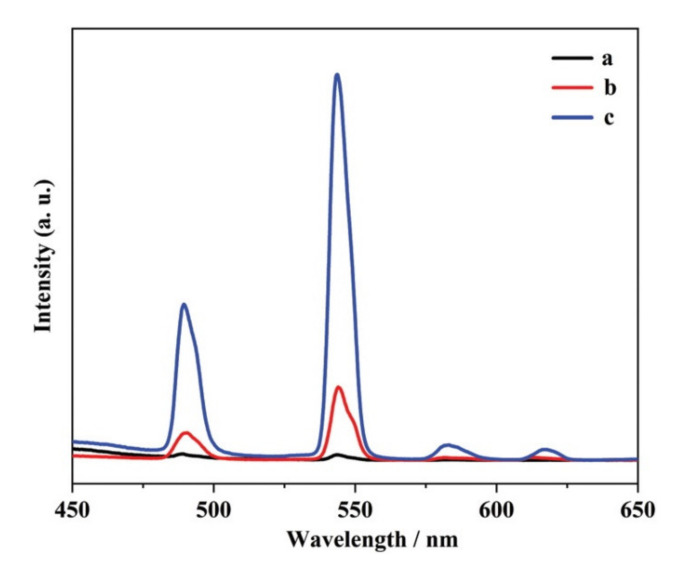
Emission spectra of (a) Tb(TODGA)_3_Cl_3_, (b) NH_4_[Tb(hfa)_4_], and (c) [Tb(TODGA)_3_][Tb(hfa)_4_]_3_ in 1-mM acetonitrile solutions. Reproduced with permission from [72].

Wu and Shen, in 2019, reported a new type of magnetic ionic liquid (MIL), incorporating the same Ln ion in both the cationic and anionic parts of the Ln-IL, with the general formula [Ln(TODGA)_3_][Ln(hfa)_4_]_3_ (Ln = Tb, Dy, Ho, Er, Tm, Yb) [72]. It is worth noting that these are the only examples of Ln-ILs with Ln-based cations. Although this was a report focusing on the magnetic properties of the Ln-ILs, the authors described the Tb-IL’s luminescence properties. Upon excitation at 352-nm UV light, the [Tb(TODGA)_3_][Tb(hfa)_4_]_3_ ionic liquid presents the common green luminescence of the Tb^3+^ ions. By comparison with the luminescence spectra of the isolated cationic and anionic components—Tb(TODGA)_3_Cl_3_ and NH_4_[Tb(hfa)_4_] (Figure 13)—where the Tb(TODGA)_3_Cl_3_ presented very low emission intensity, the Tb-IL presented the highest luminescence emission. As such, the authors concluded that the luminescence emission of the Tb-IL is mainly derived from the anionic part, and that the hfa ligand is more effective than TODGA in the sensitization of Tb^3+^ ions.

Table 2 presents a list of the Ln-ILs discussed in this review, ordered by Ln^3+^ center along with their lifetimes, associated excited levels, and physical states/transitions.

## 4. Overlook and Future Perspectives

To obtain highly efficient Ln molecular light-conversion devices, it is necessary to optimize several parameters: avoid self-quenching channels, use chromophores with high molar absorbance and ideal energy positions of singlet and triplet states (for an efficient energy transfer to Ln^3+^ ions), while avoiding competitive non-radiative pathways such as multiphonon relaxation to high-energy vibrations (e.g., O–H, C–H, and N–H stretching modes). With this context in mind, the combination of lanthanides and ionic liquids began by using ILs as matrices to protect Ln^3+^ ions from vibration-induced deactivation processes—mainly from the ever-present water adsorbed in organic solvents. Although ILs proved to provide good protection against the presence of water within the first and/second coordination spheres of the Ln centers, due to the low solubility of the Ln salts, this method usually enabled low concentrations of Ln ions, although higher concentrations could be achieved by the use of the same anionic moieties as both the ligand (of the Ln complex) and the anion (of the ILs). This shortcoming was circumvented by preparing Ln-based ionic liquids, either via direct preparation by metathesis, or by dissolving Ln salts in ILs with anions with coordinating capabilities. Ln-containing ionic liquids proved to be promising materials because, although liquids, they provide a low-phonon environment for the Ln^3+^ center, leading to appreciable excitation state lifetimes. Typically, liquid-state lanthanide compounds present lower emission quantum yields (Φ) than those in the solid state, due to a less rigid environment and energy loss from collisions. As such, it is not surprising that emission quantum yields for Ln@ILs are very low. The same reasoning is applicable to Ln-ILs although, surprisingly, out of the 58 Ln-ILs presented here, only one RTIL—[C_6_mim]_3_[Sm(NO_3_)_6_]—had its emission quantum yield determined, with a value of 2.73% [62]. Another important aspect to stress is that since Ln@ILs are liquids, no structural characterization was available for the majority of these compounds.

It is worth mentioning that many of the Ln-ILs were studied not only as phosphors, but also as paramagnetic liquids, opening avenues for multifunctional applications. Additionally, the combination of Ln and ILs has aroused so much interest that it led to the emergence of a new field of research, focused specifically on soft materials. In this area, new ionogels have been developed through covalently grafting—or simply dispersing—Ln complexes into silica-based materials, polymer matrices, liquid crystals, etc.

**Figure 14:**
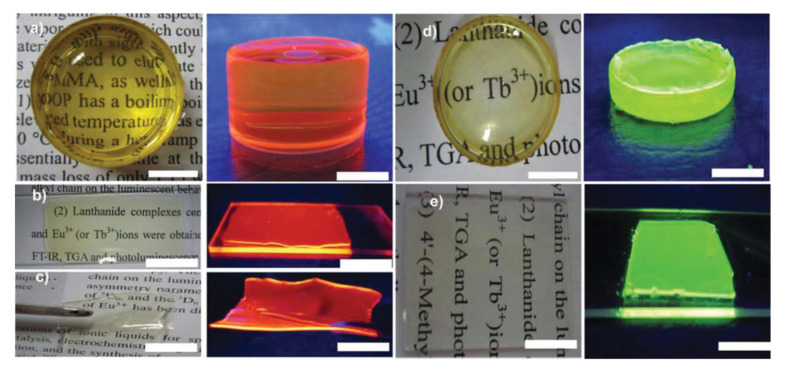
Digital photos of the Ln-ILs-PMMA: (**a**–**c**) Eu(tta)–[Carb-mim][Tf2N]@PMMA under daylight (right) and UV light (left); (**d**–**e**) Tb(sal)–[Carb-mim][Tf2N]@PMMA under daylight (right) and UV light (left). The scale bar is 1.0 cm. Reproduced with permission from [73].

It was not intended to include ionogels in this review but, just as an example, a simple and environmentally friendly (solvent-free) preparation of ionogels via the incorporation of Ln-ILs within poly(methyl methacrylate) (PMMA) was reported by Wang et al. as early as 2013 [73]. In that work, the ILs Tb(sal)@[Carb-mim][Tf_2_N] and Eu(tta)@[Carb-mim] [Tf_2_N] were directly dissolved into MMA monomers with azodiisobutyronitrile (polymerization initiator), with stirring at 80 °C, yielding a yellowish liquid that was then cast into glass slides or glass bottles. After drying, ionogels in the form of monoliths, films, and flexible self-standing films could be obtained (Figure 14).

The accomplishments described in this review have proven Ln-ILs to be outstanding and promising optical materials. However, this field of research is still underdeveloped when compared with other fields of ionic liquid chemistry. Therefore, new studies focusing on different combinations of Ln ions and new ligands will certainly lead to more efficient luminescent molecular devices, paving the way for practical applications as varied as catalysis, biochemical analysis, energy production, and non-invasive diagnostics, such as biolabels.

## Figures and Tables

**Table 1:** List of Ln@ILs and their lifetimes and physical states/transitions.

Ln@ILs	Lifetimes (Excited Level, Decay Lifetime)	Absolute Quantum Yield (%)	Ref.
Pr(Tf_2_N)_3_@[bmpyr][Tf_2_N]	^3^P_0_, 60.0 ns ^1^D_2_, 1.8 µs	0.00131	48
PrI_3_@[bmpyr][Tf_2_N]	^3^P_2_	‒	39
Pr(Tf_2_N)_3_@[bmpyr][Tf_2_N]	^3^P_2_	‒	39
Nd(Tf_2_N)_3_@[bmpyr][Tf_2_N]	^4^F_3/2_; 25.3 µs	‒	48
LaF_3_:5%Nd^3+^:betaine@[C_4_mpyr][Tf_2_N]	^4^F_3/2_	‒	43
LaF_3_:5%Nd^3+^:betaine@[C_4_mpyr][TfO]	^4^F_3/2_	‒	43
LaF_3_:5%Nd^3+^:betaine@[C_4_mim][Tf_2_N]	^4^F_3/2_	‒	43
Nd(tta)@ [3-5-carb-mim][Br]	^4^F_3/2_	‒	46
Nd(tta)@ [3-5-carb-mim][Br]	^4^F_3/2_	‒	46
[Nd(tta)_3_(phen)]@ [C_12_mim][Cl]	^4^F_3/2_; 1.52 µs	‒	42
NdI_3_@[C_12_mim][Tf_2_N]	^4^F_3/2_; 15.3 µs	1.05	40
Nd(TfO)_3_@[EMIM][TfO]	^4^G_5/2_; 376 ns	‒	25
Nd(TOS)_3_@[EMIM][TOS]	^4^G_5/2_; 800 ns	‒	25
NdBr_3_@[HMIM]Br]	^4^G_5/2_; 1510 ns	‒	25
Nd(PBS)_3_(Phen)@[HMIM](PBS]	^4^G_5/2_; 260 ns	‒	25
Nd(NTA)_4_@[HMIM]Br]	^4^G_5/2_; 1400 ns	‒	25
Sm(Tf_2_N)_3_@[bmpyr][Tf_2_N]	^4^G_5/2_; 259 µs	0.060	48
SmBr_3_@[C_12_mim]Br	^4^G_5/2_; -9°C 105°C	‒	44
[C_6_mim][Sm(tta)_4_]@[C_6_mim][Tf_2_N]	^4^G_5/2_; 78 µs	1.60	38
[C_6_mim][Sm(nta)_4_]@[C_6_mim][Tf_2_N]	^4^G_5/2_; 66 µs	1.37	38
[C_6_mim][Sm(hfa)_4_]@[C_6_mim][Tf_2_N]	^4^G_5/2_; 72 µs	1.06	38
[choline]_3_[Sm(dpa)_3_]@[C_6_mim][Tf_2_N]	^4^G_5/2_; 61 µs	‒	38
[P_8,8,8,1_][Sm(dbm)_4_]@ [P_8,8,8,1_][Tf_2_N]	^4^G_5/2_, 19.1 μs	0.6	53
TbCl_3_@[P_8,8,8,1_][BrMA]	^5^D_4_	‒	52
Tb_4_O_7_@[Carb-C_n_minm]Br	^5^D_4_	‒	51
TbBr_3_@[C_12_mim]Br	^4^D_4_; 3.4 ms	‒	44
TbCl_3_@BMIBr	^5^D_4_	‒	47
TbCl_3_(Phen)_2_(H_2_O)_3_@[C_12_mim][Cl]	^5^D_4_	‒	45
TbI_3_@[C_12_mim][Tf_2_N]	^5^D_4_	‒	41
Tb(pybox)_3_@[dppim] [bis(trifluoromethylsulfonyl)imide]	^5^D_4_, 1.22 ms	97.22	54
[C_2_mim][Tb(DCA)_4_(H_2_O)_4_] @[C_2_mim][DCA]	^5^D_4_, 0.60 ms (RT) ^a^, 0.71 ms (LT) ^b^	‒	55
Dy(Tf_2_N)_3_@[bmpyr][Tf_2_N]	^4^F_9/2_; 244 µs	0.122	48
DyBr_3_@[C_12_mim]Br	^4^F_9/2_; 53 µs	‒	44
DyI_3_@[C_12_mim][Tf_2_N]	^4^F_9/2_; 63 µs	‒	41
[C_2_mim][Dy(DCA)_4_(H_2_O)_4_] @[C_2_mim][DCA]	^4^F_9/2_, 11.9 μs (RT)	‒	55
Er(Tf_2_N)_3_@[bmpyr][Tf_2_N];	^4^S_3/2_, 0.15 µs ^4^I_13/2_, 70.6 µs	‒	48
Er(tta)@ [3-5-carb-mim] [Br]	^4^I_13/2_	‒	46
Er(tta)@ [3-5-carb-mim] [Br]	^4^I_13/2_	‒	46
Er(tta)_3_(phen)]@[C_12_mim][Cl]	^4^I_13/2_; 1.95 µs	‒	42
ErI_3_@[C_12_mim][Tf_2_N]	^4^I_13/2_; 10.4 µs	‒	40
Tm(Tf_2_N)_3_@[bmpyr][Tf_2_N]	^1^G_4_, 12.0 µs ^1^D_2_, 6.3 µs	0.209	48
[Yb(tta)_3_(phen)]@ [C_12_mim][Cl]	^2^F_5/2_; 12.4 µs	2.1	42

^a^ RT: room temperature; ^b^ LT: low temperature

**Table 2:** List of Ln-ILs and their published lifetimes and physical state/transitions.

Ln-ILs	Lifetimes (Excited Level, Decay Lifetime)	Physical State/Transition (°C)	Ref.
[C_10_mim][CeBr_6_]	‒	m.p. ^A^: 85	65
[C_4_mim]_3_[CeBr_3_Cl_3_]	‒	m.p.: 100	65
[C_4_mim][Nd(tta)_4_]	‒	RTIL	57
[P_66614_]_3_[NdCl_6_]	^4^F_3/2_, 2.470 μs	m.p.: –45.5	69
[P_4448_]_3_[NdCl_6_]	^4^F_3/2_, 2.470 μs	m.p.: 46.4 Tg: –25.5	69
[P_4444_]_3_[NdCl_6_]	^4^F_3/2_, 1.575 μs	m.p.: 65.3	69
[C_2_mim]_x–3_[Sm(NCS)_x_(H_2_O)_y_]	x=6, ^4^G_5/2_, ~23 μs	RTIL	59
	x=7, ^4^G_5/2_, ~45 μs		
	x=8, ^4^G_5/2_, ~75 μs		
[C_n_mim]_3_[Sm(NO_3_)_6_]	n= 2, 4, 8, ^4^G_5/2_, [114.4–130.3] μs n=6, ^4^G_5/2_, 114.8/3.75^α^ μs	n=2, m.p.: 82, Tg: ^B^ –49 n=4, RTIL, Tg: –38 n=6, RTIL, Tg: ~41 n=8, RTIL, Tg: –45	62
[C_4_mim]_3_[SmCl_6_]	^4^G_5/2_, 54 μs ^β^	^φ^	63
[C_4_mim][Sm(NO_3_)_4_]	‒	RTIL	64
[P_44414_]_3_[SmCl_6_]	^4^G_5/2_, 33 μs	m.p.: –44.9	69
[P_4448_]_3_[SmCl_6_]	^4^G_5/2_, 57 μs	m.p.: 47.3 Tg: –23.1	69
[P_4444_]_3_[SmCl_6_]	^4^G_5/2_, 26 μs	m.p.: 58.8	69
[RC_n_mim]_2_[Tb(NO_3_)_5_]	R=CH_3_, n=1; ^5^D_4_, 1.279 ms^#^ R=H, n=2; ^5^D_4_, 1.294 ms R=H, n=4; ^5^D_4_, 1.270 ms R=H, n=6; ^5^D_4_, 1.262 ms R=H, n=8; ^5^D_4_, 1.283 ms	m.p.: 101 RTIL, Tg: –47 RTIL, Tg: –52 RTIL, Tg: –52 RTIL, Tg: –50	30
Tb(IL-1)_3_(H_2_O)_3_	λ_exc_=370 nm, ^5^D_4_, 0.7712 ms λ_exc_=350 nm, ^5^D_4_, 0.6988 ms	RTIL (Newtonian fluid)	31
[C_12_mim]_3_[TbBr_6_]	^5^D_4_, 3.7 ms	S-LC^C^ (heating): –6 LC-S^D^ (cooling): –13 LC-L_ISO_^E^ (heating): 100.7 L_ISO_-LC^F^ (cooling): 99.4	51
6[C_12_mim]Br•[C_12_mim]_3_[TbBr_6_]	^5^D_4_, 3.3 ms	S-LC (heating): –3.5 LC-S (cooling): –18 LC-L_ISO_ (heating): 101.5 L_ISO_-LC (cooling): 100.9	51
[C_12_mpyr]_3_[TbBr_6_]	^5^D_4_, 4.0 ms	LC-L_ISO_ (heating): ~165 L_ISO_-LC (cooling): ~135	51
6[C_12_mpyr]Br•[C_12_mpyr]_3_[TbBr_6_]	^5^D_4_, 4.4 ms	S-LC (heating): 54.2 LC-S (cooling): ~30 LC-LC^G^ (heating): ~81, ~100 LC-L_ISO_ (heating): 119 LC-LC (cooling): ~119, ~78 L_ISO_-LC (cooling): ~75	51
[P_66614_]_3_[TbCl_6_]	^5^D_4_, 0.692 ms	m.p.: –49.1	69
[P_4448_]_3_[TbCl_6_]	^5^D_4_, 0.471 ms	Tg: –25.1	69
[P_4444_]_3_[TbCl_6_]	^5^D_4_, 0.416 ms	m.p.: 51.6	69
[Tb(TODGA)_3_][Tb(hfa)_4_]_3_	‒	RTIL, Tg: –31.05	72
[RC_n_mim]_2_[Dy(NO_3_)_5_]	R=CH_3_, n=1; ^4^F_9/2_, 54.75 μs^#^ R=H, n=2; ^4^F_9/2_, 59.27 μs R=H, n=4; ^4^F_9/2_, 58.17 μs R=H, n=6; ^4^F_9/2_, 58.21 μs R=H, n=8; ^4^F_9/2_, 58.56 μs	m.p.: 109 m.p.: 20, Tg: –53 m.p.: 20, Tg: –55 RTIL, Tg: –55 RTIL, Tg: –55	30
[C_6_mim]_5–x_[Dy(SCN)_8–x_(H_2_O)_x_]	x=2, ^4^F_9/2_, 23.8 μs	RTIL	56
	x=1, ^4^F_9/2_, 40.34 μs		
	x=0, ^4^F_9/2_, 48.4 μs		
[C_12_mim]_3_[DyBr_6_]	room temp., ^4^F_9/2_, 47 μs	LC-LC (heating): 27.8, 49.9, 87.3, 112.3, 115.5. LC-LC (cooling): 25.7, 83.6, 112.5, 113.5. Tg: ~20	60
	70 °C, ^4^F_9/2_, 46 μs		
[Ph_4_P]_5_[Dy(SCN)_8_]	‒	m.p.: ~35	61
[Ph_3_PBnOEt]_5_[Dy(SCN)_8_]	‒	m.p.: ~40	61
[Ph_3_PBnNO_2_]_5_[Dy(SCN)_8_]	‒	m.p.: ~45	61
[Ph_3_PBn]_5_[Dy(SCN)_8_]	‒	m.p.: ~40	61
[C_4_mim]_3_[DyCl_6_]	^4^F_9/2_, 58 μs ^β^	^φ^	63
[C_4_mim][Dy(NO_3_)_4_]	‒	RTIL	64
[P_66614_]_3_[DyCl_6_]	‒	m.p.: –47.7	69
[P_4448_]_3_[DyCl_6_]	^4^F_9/2_, 56 μs	m.p.: 3.3 Tg: –31.4	69
[P_4444_]_3_[DyCl_6_]	^4^F_9/2_, 55 μs	m.p.: 42.9	69
[MOEmim][Dy(NO_3_)_4_]	‒	RTIL	71
[Ho_5_(C_2_H_5_-C_3_H_3_N_2_-CH_2_COO)_16_(H_2_O)_8_](Tf_2_N)_15_	^5^F_5_, 0.8 μs	metastable RTIL Tg^Δ^	2
[C_4_mim]_3_[ErCl_6_]	^4^I_13/2_, 2.5 μs ^β^	^φ^	66
[P_66614_]_3_[ErCl_6_]	^4^I_13/2_, 2.725 μs	m.p.: –40.4	69
[P_4448_]_3_[ErCl_6_]	^4^I_13/2_, 2.736 μs	m.p.: –48 Tg: –67.9	69
[P_4444_]_3_[ErCl_6_]	^4^I_13/2_, 2.066 μs	m.p.: 51.3	69
[Er_5_(C_2_H_5_-C_3_H_3_N_2_-CH_2_COO)_16_ (H_2_O)_8_](Tf_2_N)_15_	^4^I_13/2_, 0.6 μs^Ø^	m.p.: 74.6 (metastable RTIL) Tg^Δ^	2
[C_4_mim]_3_[YbCl_6_]	^2^F_5/2_, 19.7 μs ^β^	^φ^	66

^A^ m.p.: melting point (measured on heating); ^B^ Tg: glass transition; ^C^ S–LC: solid–liquid crystal transition; ^D^ LC–S: liquid crystal–solid transition; ^E^ LC–L_ISO_: liquid crystal–isotropic liquid transition; ^F^ L_ISO_–LC: isotropic liquid–liquid crystal transition; ^G^ LC–LC, liquid crystal–liquid crystal transition; ^α^ the lower value was measured after addition of water; ^β^ average lifetime for the solution and crystal phases; ^φ^ studies were made in crystal form and in C_4_mimCl solutions, but not in pure [C_4_mim]_3_[LnCl_6_] form; Tg^Δ^ several glass transitions were recorded, with the temperature of the transitions gradually changing with consecutive cycles; ^Ø^, value of lifetime measured both in liquid and solid states; ^#^ this compound is not an IL, but a low-melting-temperature salt added here for purposes of comparison.

## Acronyms

AA	Acetylacetonate
BA	Benzoate
betaine	N,N,N-trimethylglycine
BF_4_	Tetrafluoroborate
bmpyr	1-N-butyl-1-methylpyrrolidinium
BrMA	Conjugate base of bromomalonaldehyde
[Carb-mim]	3-(3-Carboxy-propyl)-1-methylimidazolium
[3-5-carb-mim]	3-(5-Carboxy propyl) -1-methylimidazolium
C_n_mim	1-Alkyl-3-methylimidazolium
C_2_mim	1-Ethyl-3-methylimidazolium
C_3_mim	1-Propyl-3-methylimidazolium
C_4_mim	1-Butyl-3-methylimidazolium
C_4_mpy	N-butyl-4-methylpyridinium
C_4_mpyr	1-Butyl-1-methylpyrrolidinium
C_6_mim	1-Hexyl-3-methylimidazolium
C_8_mim	1-Octyl-3-methylimidazolium
C_10_mim	1-Decyl-3-methylimidazolium
C_12_mim	1-Dodecyl-3-methylimidazolium
C_12_mpyr	N-dodecyl-N-methylpyrrolidinium
DCA	Dicyanamide
choline	(2-Hydroxyethyl)trimethyl ammonium
dbm	1,3-Diphenylpropane-1,3-dione
dpa	Pyridine-2,6-dicarboxylate
dppim	1,3-Bis-{3-(diphenylphosphinyl)propyl}imidazole
hfa	1,1,1,5,5,5-Hexafluoroacetylacetonate
MC_1_mim	1,2,3-Trimethylimidazolium
MIL	Magnetic ionic liquid
MOEmim	1-(2-Methoxyethyl)-3-methylimidazolium
NIR	Near-infrared
nta	Naphthoyltrifluoroacetone
P_8,8,8,1_	Trioctylmethylphosphonium
phen	1,10-Phenantroline
PMMA	Poly(methyl methacrylate)
pybox	2,6-Bis[(4R)-4-phenyl-2-oxazolinyl]pyridine
RTIL	Room-temperature ionic liquid
sac	Saccharinate
sal	Salicylate
Tf_2_N	Bis-(trifluoromethanesulfonyl)amide
TfO	Trifluoromethanesulfonate
tta	2-Thenoyltrifluoroacetonate
TODGA	N,N,N’,N’-tetra(n-octyl)diglycolamide
